# Ciguatera Fish Poisoning: The Risk from an Aotearoa/New Zealand Perspective

**DOI:** 10.3390/toxins12010050

**Published:** 2020-01-15

**Authors:** Lesley L. Rhodes, Kirsty F. Smith, J. Sam Murray, Tomohiro Nishimura, Sarah C. Finch

**Affiliations:** 1 Cawthron Institute, Halifax Street Campus, Private Bag 2, Nelson 7042, New Zealand; kirsty.smith@cawthron.org.nz (K.F.S.); sam.murray@cawthron.org.nz (J.S.M.); tomohiro.nishimura@cawthron.org.nz (T.N.); 2 AgResearch Limited, Ruakura Research Centre, Private Bag 3123, Hamilton 3240, New Zealand; Sarah.finch@agresearch.co.nz

**Keywords:** ciguatera fish poisoning, ciguatoxins, maitotoxins, *Gambierdiscus*, *Fukuyoa*, Aotearoa, New Zealand, Rangitāhua, Kermadec Islands

## Abstract

*Gambierdiscus* and *Fukuyoa* species have been identified in Aotearoa/New Zealand’s coastal waters and *G. polynesiensis*, a known producer of ciguatoxins, has been isolated from Rangitāhua/Kermadec Islands (a New Zealand territory). The warming of the Tasman Sea and the waters around New Zealand’s northern subtropical coastline heighten the risk of *Gambierdiscus* proliferating in New Zealand. If this occurs, the risk of ciguatera fish poisoning due to consumption of locally caught fish will increase. Research, including the development and testing of sampling methods, molecular assays, and chemical and toxicity tests, will continue. Reliable monitoring strategies are important to manage and mitigate the risk posed by this emerging threat. The research approaches that have been made, many of which will continue, are summarised in this review.

## 1. Introduction

Ciguatera fish poisoning (CFP) may cause neurological, gastrointestinal, and cardiological symptoms, and can be fatal [1,2]. There may also be numbness and cold allodynia (the feeling of pain at temperatures which are normally well tolerated), a symptom also experienced with exposure to brevetoxins. If the symptom is present, paraesthesia (a burning or prickling sensation) differentiates CFP from other forms of food poisoning and gastroenteritis [3,4].

CFP is caused by the consumption of fish that have sequestered ciguatoxins (CTXs), with the onset of symptoms varying between patients, from less than 1 h to 48 h following ingestion. The severity and duration of symptoms is also variable, being dependant on the quantity and identity of the CTXs consumed. This is in turn influenced by the type of fish consumed and the geographical location. In some sufferers, persistent symptoms of up to years have been reported [4]. There is currently no known reliable antidote, although Octopus Bush (*Heliotropium foertherianum*, Boraginaceae) is commonly used in French Polynesia (refer https://en.ird.fr).

The sequestration of toxins occurs when herbivorous fish consume toxin-producing epiphytic dinoflagellates in the genus *Gambierdiscus* R.Adachi & Y.Fukuyo while feeding on macroalgae or during scraping of corals, for example, maito (*Ctenochaetus striatus* family Acanthuridae) and parrot fish (family Scaridae), respectively. Larger carnivorous fish sequester toxins by consuming the smaller toxic fish, and humans eating the contaminated fish may become ill even after lengthy cooking as heat will not destroy the toxins [3]. The global distribution of species in the genus *Gambierdiscus*, and the closely related genus *Fukuyoa* F.Gómez, D.X.Qiu, R.M.Lopes, & Senjie Lin (previously classified as *Gambierdiscus*), has been summarised for a ‘HAB-MAP’ project (in which harmful or toxic species have been mapped by region) in collaboration with the International Society for the Study of Harmful Algae (ISSHA). The data, including toxin profiles, are hosted by the Ocean Biogeographic Information System (OBIS (http://haedat.iode.org/) [5].

In recent years, CFP has also been linked to consumption of gastropods contaminated with CTXs [6,7]. The commonly named ‘commercial top shell’ (*Tectus niloticus* Tegulidae, Gastropoda), caused a major illness event on Nuku Hiva Island, in the Marquesas Archipelago, French Polynesia, in 2014. The CTXs producer *G. polynesiensis* Chinain & M.A.Faust dominated the *Gambierdiscus* communities present at the time [6]. In addition to the gastropod *T. niloticus*, CTXs have also been reported in a bivalve mollusc (the giant clam *Tridacna maxima* Tridacnidae, Bivalvia) and a sea urchin (*Tripneustes gratilla* Toxopneustidae, Echinoidea). These findings, which have been summarised in a review by Chinain et al. [8], compound the risks for the many small Pacific Island communities that are dependent on the reef system for sustenance and trade. The known number of vectors of CTXs are also increasing as investigations into CFP increase. For example, starfish (*Ophidiaster ophidianus* Ophidiasteridae and *Marthasterias glacialis* Asteriidae, Class Asteroidea), collected off the coasts of Madeira and Morocco have been identified as potential vectors for human intoxications [9].

CFP is the most frequently reported fish-related poisoning in humans and is a significant health problem in the Pacific region [10], although with growing global demands for seafood and increasing pathways to market, CFP is increasingly an international issue [4]. During the last decade there has been an increase in reports of New Zealand tourists returning from the Pacific Islands and northern Australia with illnesses linked to the consumption of reef fish, and many of these illnesses have been diagnosed as CFP [3,11,12,13]. In New Zealand, between 2006 and 2014, there were fifty-four hospitalisations diagnosed as CFP. One cluster of cases was attributed to the consumption of imported Moray eel (family Muraenidae), which was positively confirmed as containing Pacific-CTX-1B (P-CTX) [14]. No illnesses resulting from the consumption of locally caught fish have been formally documented for New Zealand or Rangitāhua/Kermadec Islands.

In Australia, CFP cases have been mainly linked to eating CTXs-contaminated Spanish mackerel (*Scomberomorus commerson* Lacepède), a pelagic fish species. Tropical reef fish have, however, been implicated in Queensland intoxication events. More than a thousand CFP cases have been reported in Australia over the last four decades, including two fatalities, but since the reporting rate is thought to be extremely low, this perhaps represents only 10% of actual cases [15,16]. While most Australian cases have occurred in Queensland, there have also been an increasing number of reports from New South Wales over the last five years, with the analogue P-CTX-1B reported in remnant samples from consumed and locally caught Spanish mackerel [17].

As the genera *Gambierdiscus* and *Fukuyoa* have both now been identified in New Zealand waters [11,18], it is only a matter of time before potential CTXs producers proliferate in New Zealand’s northern subtropical waters (Northland), increasing the risk of CFP occurring locally. Monitoring and research are therefore vitally important to understand and mitigate the risk posed by this emerging threat.

## 2. Distribution and Habitat of *Gambierdiscus* and *Fukuyoa*

### 2.1. Global Distribution

The current focus on CFP internationally has meant that there has been a rapid rise in new *Gambierdiscus* species descriptions, with a 30% increase between 2016 and 2017 alone [10,12] and additional species being described in the last two years. There are currently eighteen published species of *Gambierdiscus* and three described *Fukuyoa* species, plus one putative species (Table 1). Three species have been identified as potential producers of CTXs or CTX-like compounds: CTXs production has been confirmed for *G. polynesiensis* [19] by liquid chromatography tandem mass spectrometry (LC-MS/MS). *G. silvae* Fraga & Rodriguez [20] and *G. excentricus* Fraga [21,22,23,24] were reported as positive for CTXs using the neuro-2a assay, with *G. silvae* being highly toxic in this assay (2.1–4.8 pg Caribbean-CTX-1 equivalents; C-CTX) [20,21].

**Figure 1:**
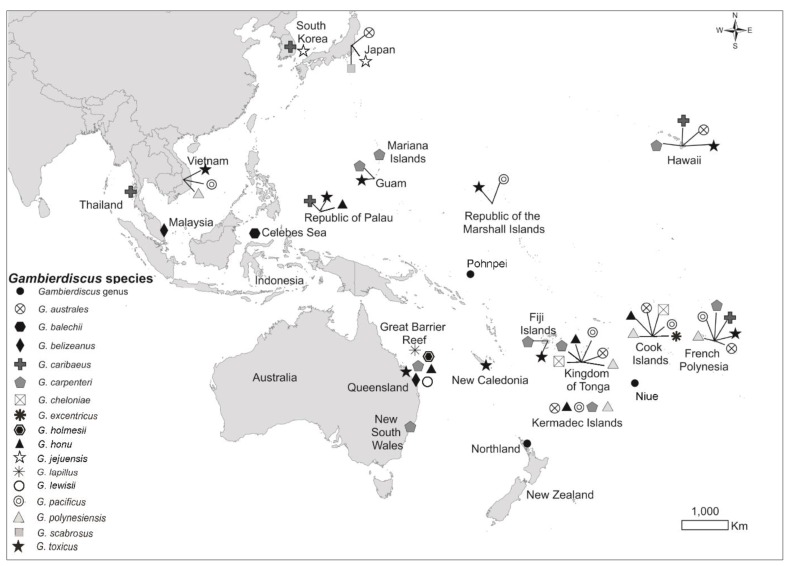
Map of Pacific region showing the geographic distribution of *Gambierdiscus* species. (Modification of Figure 3, Rhodes et al. 2017 [28], 2017 *Harmful Algae News*).

Over the last decade these species have been reported not only from tropical and subtropical waters, but also from more temperate regions, including the coastal waters of New South Wales, Australia [20,25,26,27,28], and additional species have been reported from more sites throughout the Pacific region (Figure 1) [6,13,23]. Of the eighteen described *Gambierdiscus* species, only *G. silvae* and *G. carolinianus* have not been reported in the Pacific region. One concern is the potential for ‘tropicalisation’ of temperate marine ecosystems, which could occur in Australasia (including Northland, Aotearoa/New Zealand), where average coastal seawater temperatures are rising and where currents are dispersing those warmer waters [25,29,30].

### 2.2. Species of Gambierdiscus and Fukuyoa Isolated from New Zealand and Rangitāhua/Kermadec Islands

**Figure 2:**
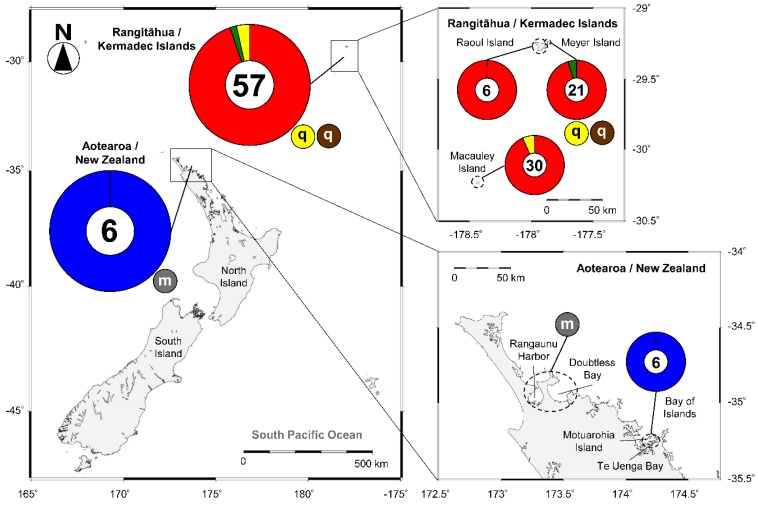
Summary of the geographic distribution of the genera *Gambierdiscus* and *Fukuyoa* in coastal areas of Rangitāhua/Kermadec Islands and Aotearoa/New Zealand. The numbers in each pie chart indicate the number of strains identified by DNA sequencing. The letters in each circle indicate other detection methods (q: quantitative PCR; m: light microscopy). Colours represent *Gambierdiscus* and *Fukuyoa* species (red: *G. australes*; green: *G. honu*; yellow: *G. polynesiensis*; brown: *G. pacificus*; blue *F. paulensis*; grey: *G.* cf. *toxicus*).

The results of repeated sampling in both the northern and southern groups of Rangitāhua/Kermadec Islands, a New Zealand territory 1000 km north east of New Zealand [11,12], confirm that *Gambierdiscus* species are present throughout the oceanic archipelago of islands (Figure 2). *Gambierdiscus* cells have been isolated from samples collected from macroalgae in the northern, largest, and volcanically active Raoul Island and from nearby North Meyer Island. *Gambierdiscus* has also been isolated from Macauley Island (100 km SSW of Raoul Island and the second largest island) [31]. Species isolated include *G. honu* Rhodes, Smith, & S.A.Murray (first described from Macauley Island [12]), *G. australes* Chinain & M.A.Faust, and *G. polynesiensis* (Table 1, Figure 2).

Sampling in subtropical northern New Zealand waters has confirmed that *Fukuyoa* is well established in Northland. It has been repeatedly detected in samples collected in the Bay of Islands, at Te Uenga Bay and Motuarohia Island [32,33]. The first isolation was from samples collected in February 2013, when it was reported as *Gambierdiscus* cf. *yasumotoi* M.J.Holmes. The genus, which is more globular in shape than the related lenticular genus *Gambierdiscus*, has since been reclassified as *Fukuyoa* [34]. The Northland isolate was confirmed as *F. paulensis* Gómez, Qiu, Lopes, & Lin and has been detected in samples collected on artificial substrates by high-throughput sequencing (HTS) combined with DNA barcoding methodologies (metabarcoding), as well as by phylogenetic analyses of isolated and cultured cells [35]. The latest isolates of *F. paulensis* were obtained during a sampling trip in February 2019 (L. Rhodes, Cawthron Institute, unpublished data) and have been deposited in the Cawthron Institute Culture Collection of Microalgae (CICCM; codes CAWD306 and CAWD308). Identification was confirmed by phylogenetic analyses of DNA sequence data. To date, only one observation of a *Gambierdiscus* cell, using light microscopy, has been reported in New Zealand. This was from a sample collected from the floating macroalga *Sargassum* Agardh (Phaeophyta) collected in Northland waters in the vicinity of Doubtless Bay [18] (Table 1, Figure 2). The species was reported in 1996 as *G. toxicus* Adachi & Fukuyo, but identifications made by microscope examination alone prior to 2009 are uncertain due to the similar morphologies and morphological plasticity of some species [10,11,36]. Molecular markers now allow rapid and robust determinations (see Section 4).

### 2.3. Macroalgae Substrates

Many of the samples collected from Rangitāhua/Kermadec Islands were from mixed red macroalgal assemblages (Rhodophyta) shaken into 50 mL tubes containing ambient seawater [12]. In the case of *G. honu*, cells were isolated from the material released after brushing non-geniculate coralline turfs [39,40]. The more commonly occurring *Gambierdiscus* species, *G. australes*, was also found on the rhodophytes *Spyridia filamentosa* (Wulfen) Harvey, *Asparagopsis taxiformis* (Delile) Trevis, and *Dasya baillouviana* (Gmelin), and on the chlorophytes *Microdictyon umbilicatum* (Velley) Zanardini and *Caulerpa webbiana* Mont. [37]. During a later expedition to Rangitāhua in 2018, *G. australes* was isolated from samples collected from the phaeophyte *Dictyota* J.V.Lamouroux, but at that same time no dinoflagellates were found in samples isolated from the co-occurring rhodophyte *Delisea pulchra* (Greville) Montagne [39].

Studies by Parsons et al. [41] and Rains and Parsons [42] described the different behaviours exhibited to different host macroalgae substrates by different species of *Gambierdiscus* and *Fukuyoa.* Attachment and detachment, for example, appeared to be altered in the presence of some macroalgae species. *Gambierdiscus* is not considered to be an obligate epiphyte and there is evidence to suggest that different macroalgae species may stimulate or inhibit the dinoflagellate’s growth, possibly through macroalgal exudates. There is some further evidence for allelopathic interactions between *G. carpenteri* Kibler, Litaker, M.A.Faust, W.C.Holland, Vandersea, & P.A.Tester and co-occurring epiphytic dinoflagellates impacting on growth and for these interactions to be affected by temperature [43]. In other studies carried out in the Rarotongan lagoons, Cook Islands (approximately 2000 km northwest of the Kermadec Islands), *G. honu* and *G. cheloniae* Smith, Rhodes, & Murray [44] were isolated from the phaeophyte *Turbinaria* J.V.Lamouroux and *G. cheloniae* was also isolated from the rhodophyte *Jania* J.V.Lamouroux. The co-occurring species, *G. australes* and *G. pacificus* Chinain & M.A.Faust were isolated from the chlorophyte *Halimeda* sp. J.V.Lamouroux and *G. australes* was also isolated from the chlorophyte *Cladophora* Kützing and the phaeophyte *Padina* Adanson [33,44,45,46]. *Gambierdiscus polynesiensis* was only isolated from mixed rhodophytes in the Cook Islands during those sampling efforts [46].

A study of the growth and epiphytic behaviour of three *Gambierdiscus* species, *G. balechii, G. caribaeus* Vandersea, Litaker, M.A.Faust, Kibler, W.C.Holland, & P.A.Tester, and a new *Gambierdiscus* ribotype, type 7, supported the contention that there are no specific preferences for macroalgal host species and that other environmental factors, such as light avoidance, may be at play [47]. Further research is needed to identify environmental factors that influence macroalgae composition and distribution, and thus, an increased risk of CFP [48].

In Northland, New Zealand, *Fukuyoa* has been isolated most often from areas where coralline macroalgae dominate, in particular *Jania rosea* (Lamarck) Decaisne [32]. It is therefore apparent that while *Fukuyoa* in New Zealand has been found typically attached to corallines, *Gambierdiscus* can live epiphytically on a variety of macroalgae, eel grasses [27] and (particularly dead) coral substrates [49]. Future sampling should therefore take this into consideration.

## 3. Field Methods for Sampling Microalgae and CFP Toxins

### 3.1. Artificial Substrate Samplers for Microalgae 

Cells collected from macrophytes are generally reported in terms of cells per gram wet weight of macroalgae or seagrass. This unit can be compared easily between different host species but does not account for the surface area of the macrophyte being sampled, an important factor determining how many cells macrophytes may host. An artificial substrate offers the advantage of comparing fixed surface areas. A disadvantage is the need for those sampling (often divers dependent on the prevailing weather conditions) to return to the same site twice during a twenty-four hour period [50].

The genus *Gambierdiscus* is not considered an obligate epiphyte and has varied degrees of motility depending on the available macroalgal substrates. Cells can also be resuspended due to mechanical action, such as wave action or anthropogenic disturbances [41]. It is therefore feasible that the dinoflagellate could attach to other artificial substrates. A study by Tester et al. [51] showed a direct correlation between benthic/epiphytic dinoflagellates on the naturally occurring macroalgae and on the artificial substrate sampler used (black fibre glass screen), with equilibration occurring over 24 h. The sampler was considered the first step towards developing a standardised sampling method for potentially harmful benthic and epiphytic dinoflagellates, and towards a cell-based monitoring programme for CFP [51]. A similar sampler was constructed for use in the Bay of Islands, Northland, Aotearoa/New Zealand, and was found to be particularly useful for obtaining live dinoflagellate cells free from debris for microscopic analysis and molecular identification methods [35].

The New Zealand samples were collected in a protected embayment, but some caution in other environments has been advised by Parsons et al. (2017) [52]. In the Florida Keys, the artificial substrates tested (including the black fibre glass screen) were deployed at four different sites for a month. The length of time deployed does bring other factors into play, not least the loss of some of the artificial substrates. The usefulness for collection of benthic/epiphytic dinoflagellates was acknowledged, but the limitations of the method for quantification were noted due to the wide variation in the slopes of pairings of macroalgae and artificial substrates that had significant correlations [52]. In another study carried out at fifteen sites in Tonga [50,53], the correlation between natural and artificial substrates was good, except for one site, Ha’ateiho, which was a high energy area. At this site *Gambierdiscus* was detected in samples collected from macrophytes but not from the artificial sampler [50]. The artificial substrate method was also trialled in Macronesia and the results supported its use for monitoring of benthic HABs [54].

New methods are being trialled, for example the benthic dinoflagellate intergrator (BEDI) [55], which allows an assessment of relative abundance of the epiphytic dinoflagellate *Ostreopsis* J.Schmidt (Ostreopsidaceae) in both the biofilm and surrounding water, although limitations of this method are acknowledged. An internationally accepted and reliable method of assessing CFP risk is highly desirable.

### 3.2. Sampling for Toxins In Situ

One drawback for monitoring programmes based on microalgal detection is that false alarms may be raised if nontoxic *Gambierdiscus* species or nontoxic strains of known toxin producers are reported [55]. Toxins can be sampled directly from the environment and one approach is solid phase adsorption toxin tracking (SPATT) devices [56]. The analogues P-CTX-3B, P-CTX-3C, and 44-methylgambierone (44-MG), but not maitotoxin-1 (MTX-1), were detected by LC-MS/MS after 48 h deployment of the resin at Nuku Hiva Island, French Polynesia [57]. While this approach offers a potential method for CFP risk assessment in the environment, a disadvantage is that toxin levels retained on the resin cannot be easily converted to toxin concentrations in the environment unless water flow measurements are conducted simultaneously. It is, however, a promising method that is being further trialled.

## 4. Molecular Methods for Detection and Quantification of *Gambierdiscus* and *Fukuyoa*

The differentiation of *Gambierdiscus* and *Fukuyoa* cells to species level using light microscopy is difficult, and in some cases impossible. Scanning electron microscopy (SEM) is time consuming and, due to the high purchase cost of the equipment, is not always available. SEM also requires reasonably clean samples and good cell numbers.

In recent years quantitative polymerase chain reaction (qPCR) methods have been successfully used for the rapid determination of which species are present at a chosen site. The suite of assays available now covers most of the species described, including an assay that can detect all species of *Gambierdiscus* and *Fukuyoa* genera [58]. Vandersea et al. [59] developed species-specific semi-qPCR assays for the detection and enumeration of *G. belizeanus* Faust, *G. caribaeus*, *G. carolinianus* Litaker, Vandersea, M.A.Faust, Kibler, W.C.Holland, & P.A.Tester, *G. carpenteri*, and *Fukuyoa ruetzleri* (Faust, Litaker, Vandersea, Kibler, Holland, & Tester) Gómez, Qiu, Lopes, & Lin (previously known as *G. ruetzleri* Faust, Litaker, Vandersea, Kibler, Holland, & Tester) using a SYBR green format. It was the trialling of these early assays that highlighted the complexity of *Gambierdiscus* populations, with many species being present at one site. A sensitive and specific assay for *G. lapillus*, which may produce CTX congeners in Australia, has also been recently validated [60].

Semi-quantitative PCR, using ten species-specific assays, was used to determine the presence of *G. polynesiensis* in samples collected on artificial substrates from Anaho Bay, Nuku Hiva Island, French Polynesia [6]. The findings were concurrent with the determination of CTXs in the commercial top shell, *Tectus niloticus*, supporting the contention that gastropods can be vectors as well as finfish. Although *G. caribaeus*, *G. carpenteri*, *G. pacificus*, and *G. toxicus* were also detected, only *G. polynesiensis* isolates demonstrated toxicity as determined using the neuro-2a assay.

Using qPCR or PCR assays, *G. polynesiensis, G. silvae*, and *G. excentricus* (known producers of CTXs and/or CTX-like compounds) can be reliably and rapidly detected [6,61]. The latter two species are of critical importance in the Caribbean, but currently *G. polynesiensis* is considered of greatest concern in the Pacific region and it has been isolated from Rangitāhua/Kermadec Islands. From a monitoring perspective, the genus level-qPCR assay is a useful tool that can be used to screen environmental samples so that only positive samples are further analysed (for example, cell isolations, species-specific assays, and metabarcoding) [35,50].

The qPCR approach, using a TaqMan probe, has also been used successfully in Japan for the detection and quantification of toxic versus nontoxic *Gambierdiscus* species/phylotypes (toxicity determined by mouse bioassay). The species/phylotypes tested included toxic *G. australes*, toxic *G. scabrosus*, a potentially nontoxic phylotype *Gambierdiscus* sp. type 2, now described as *G. jejuensis* S.H.Jang, & H.J.Jeong [62], and a toxic phylotype, *Gambierdiscus* sp. type 3 [63]. It is expected to be a powerful tool for determining distribution patterns and for risk monitoring in coastal Japan. A reliable approach for the design of molecular assays is to target the ribosomal DNA (rDNA) regions, including the small subunit (SSU), internal transcribed spacer region (ITS), and the large subunit (LSU). Using phylogenetic methods, the D8-D10 LSU rDNA region is often applied to delineate species-level groupings, although the SSU rDNA is also useful for resolving the major *Gambierdiscus* clades [24]. Phylogenetic analyses of the SSU and LSU of *Gambierdiscus* have highlighted four clades being present within the genus, with *Fukuyoa* (previously classified as *Gambierdiscus*) forming a fifth, separate clade [64]. Discrepancies of clade resolution between the different rDNA gene regions have, however, been observed in *Gambierdiscus* [43,64].

Metabarcoding shows promise as a species-specific detection approach and has advantages over microscopic analysis, particularly when benthic/epiphytic dinoflagellate species other than *Gambierdiscus* are blooming or when disturbed sediments make counting difficult [35,58]. The identification of *Gambierdiscus* species present at a site cannot, however, provide a determination for when human health may be at risk as so many interacting factors are at play, from toxin production and transfer within the food web to habitat-damaging storms or anthropogenic disturbances [50]. In samples collected from Te Uenga Bay in northern Aotearoa/New Zealand, metabarcoding determined the presence of thirty-five dinoflagellate species, including *F. paulensis* [58]. In comparison, quantification using the light microscope determined the presence of only five dinoflagellate species (including the large cells of *Fukuyoa*) due to an *Ostreopsis* cf. *siamensis* Johs.Schmidt bloom swamping out the other less prevalent and/or smaller dinoflagellate species [35]. Surveys of benthic dinoflagellates in the Kingdom of Tonga found metabarcoding to be time efficient, and with higher taxonomic resolution than qPCR, and was used successfully to detect *Gambierdiscus* and *Fukuyoa* species in environmental samples at fourteen out of the fifteen habitat types sampled, including seagrass and mixed macroalgae communities, and at low and most high energy sites [53].

## 5. Toxin Production by *Gambierdiscus* and *Fukuyoa* Species Isolated from Aotearoa/New Zealand and Rangitāhua/Kermadec Islands

### 5.1. Ciguatera Fish Poisoning Related Toxins and Their Toxicity

**Figure 3:**
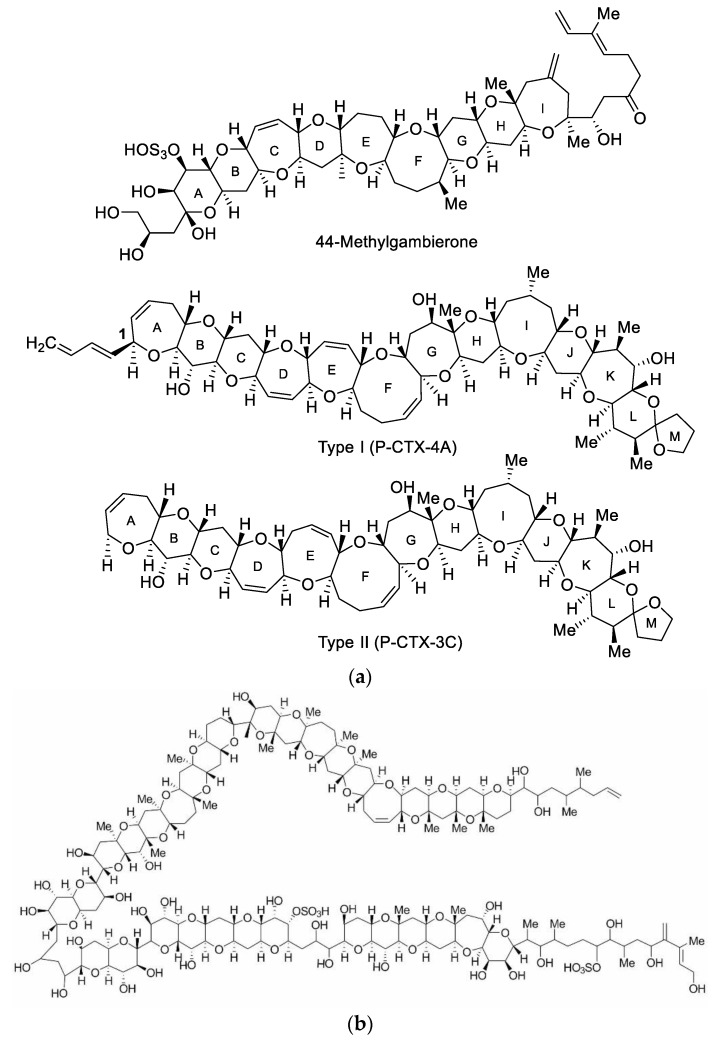
(**a**) Chemical structures of 44-methylgambierone, previously reported as maitotoxin-3 (copied from Murray et al., 2019 [73]). There are two distinct structural types in the P-CTX class: Type I (P-CTX-4A) and Type II (P-CTX-3C). Both contain a 13-ring (A–M) backbone, with the main differences being the aliphatic hydrocarbon chain on C1 (A-ring) for Type I, and the size of the E-ring (seven-membered for Type I and eight-membered for Type II). (**b**) Chemical structure of maitotoxin-1 (MTX-1: adapted from Murata et al. 1993 [71].

The CTXs are polyether ladder compounds that have a polyketide origin, suggesting that polyketide synthases (PKS) are involved in their production. Gene catalogues have been produced for the CTXs producers *G. polynesiensis* and *G. excentricus* and, in addition to a vast diversity of PKS genes being detected, a clear distinction has been made between those genes responsible for fatty acid and polyketide biosynthesis in *Gambierdiscus* [65,66]. It is likely that many genes are associated with CTXs production and dinoflagellates may also produce a variety of diverse PKS-related compounds, making it even more difficult to link specific pathways and compounds [67]. The finding that the average toxicity (Table 2) of a species may be inversely proportional to growth rate suggests that there may be an evolutionary trade-off between toxin production (possibly for defence) and growth [22]. The CTXs are potent, lipophilic, polyether toxins, which activate voltage-gated sodium channels in mammalian cells. The CTXs known to be produced by *Gambierdiscus* species include P-CTX-3B, -3C, -4A, and -4B (Figure 3A). These differ from the CTXs found in fish, as the algal CTXs are bio-transformed to form even more toxic congeners, for example, P-CTX-1B [26,68]. Roeder et al. (2010) [69] reported that 2,3-dihydroxy-P-CTX-3C was produced by *G. australes*, *G. carpenteri*, *G. caribaeus*, *G. pacificus*, and *G. toxicus*. It is noted that this analogue is considered a fish metabolite and not produced by the dinoflagellates [70].

The MTXs are also potent polyether toxins but are water soluble (Figure 3B). The MTXs increase intracellular calcium levels in mice and are one of the most lethal nonproteinaceous natural compounds known, although oral potency is lower than that by intraperitoneal injection, probably due to its hydrophilicity. MTXs are therefore most likely to contribute to CFP if gut and liver tissues of the fish are consumed [23,71,72]. Other compounds produced by *Gambierdiscus* species include gambieric acids, gambieroxide, gambierol, and gambierone [72].

The most accurate assessment of the risks posed by *Gambierdiscus* and *Fukuyoa* species to consumers would be by testing them in an in vivo toxicity assay. Traditionally this would be done using a mouse bioassay (MBA), which correlates death times of mice with toxicity. However, time to death is not a true measure of toxicity since the toxicokinetics of absorption may be different for different toxic analogues, which would affect death times but not necessarily toxicity. The MBA is also not capable of high throughput analysis and the ethics of using the assay for large numbers of samples is of significant concern. For these reasons the MBA is no longer used for routine screening and monitoring of microalgal samples, although an in vivo mouse bioassay is still used as a research tool. In this case, it is a true toxicity assay that is used to determine LD_50_s of microalgal strains and pure compounds.

There are a number of alternatives used to estimate the toxicity of microalgal strains that utilise the mechanism of action of the CTXs and MTXs. The neuro-2a mouse neuroblastoma assay (N2a) measures potency of extracts to voltage-gated sodium channels and has been used extensively in CFP research [87]. A receptor binding assay (RBA) has also been used, which is based on binding competition between CTXs-containing samples and tritiated brevetoxin, a compound which is also active on the sodium channel receptor [19]. Due to the limitations posed by the use of tritiated brevetoxin, another variation is also used with fluorometric detection rather than scintillation counting as the endpoint [88]. Another alternative is the human erythrocyte lysis assay (HELA) which measures heamolytic activity associated with the presence of MTXs [23,89]. CTXs and MTX activity can also be measured using a calcium influx SH-SY5Y cell fluorescence imaging plate reader (FLIPR) bioassay [23,79]. New approaches to determining CFP risk are being sought and one such approach is the genetic modification of yeast cells to encode specific transcriptional reporters that respond to CTXs exposure. In preliminary studies, the CTXs were not toxic to the yeast, even at concentrations of 1 µM, but activation of the calcineurin signalling pathway was observed, suggesting potential for further development of this method as a monitoring tool [90].

Risk posed by *Gambierdiscus* and *Fukuyoa* strains can also be estimated by LC-MS/MS analysis, which is currently the favoured approach for determining whether CTXs or MTXs are present in *Gambierdiscus* isolates from New Zealand and its territories [38]. However, the availability of chemical standards is an issue and currently the method is limited to the detection of characterised microalgal-derived toxins P-CTX-3B/3C, P-CTX-4A/4B, MTX-1, and 44-MG. To be able to convert the quantitative determination of CTX and MTX congeners to risk requires knowledge of the toxicity of each compound so that toxicity equivalence factors (TEFs) can be applied. It also assumes that the cause of CFP is known, which is clearly not the case for a number of reported poisoning events in which the food consumed is unavailable for testing. In reality, the risk posed by *Gambierdiscus* and *Fukuyoa* strains is assessed using a combination of the approaches described above. Due to the different lipophilicities of CTXs and MTX, fractions can be generated that split the two classes of compounds, allowing each to be tested separately. Any CTXs or MTX activity detected can then be further investigated by LC-MS/MS and toxicological evaluation.

### 5.2. Ciguatera Toxin Risk in New Zealand Waters

*Gambierdiscus polynesiensis* is the only verified producer of CTXs that has been found in New Zealand’s territorial waters. Two isolates of *G. polynesiensis* were collected from Macauley Island. One isolate (CICCM code CAWD254) produced extremely low concentrations of CTXs (including P-CTX-3B and isomers of P-CTX-3B,C and P-CTX-4A,B as determined by LC-MS/MS; unpublished data), whereas the other isolate (CAWD259) produced no CTXs at the level of detection of the analysis [12]. This is unusual as *G. polynesiensis* is a well-documented CTXs producer [19]. For example, a *G. polynesiensis* isolate from the Cook Islands (CAWD212) originally produced P-CTXs (155 pg/cell; unpublished data), with P-CTX-3B being 65% of the total CTX detected. Furthermore, this strain showed potent toxicity to mice by both intraperitoneal (i.p) injection and by gavage yielding symptoms of hypersalivation, which is characteristic of CTXs-induced toxicity [38]. All strains of *G. polynesiensis* analysed by LC-MS/MS also showed the presence of an analogue of gambierone, 44-methylgambierone (44-MG) [38]. This compound has been fully characterised by nuclear magnetic resonance spectroscopy [73] and found to be the same structure as that previously reported as MTX-3 [91,92]. Work is currently underway to determine the toxicity of 44-MG, although a review of current literature shows that there is no association between concentrations of 44-MG and acute toxicity in mice, indicating that an unknown toxin, or toxins, is contributing to toxicity for a number of *Gambierdiscus* species that have currently only been shown to produce 44-MG [38].

The only known producer of MTX-1 in New Zealand waters is *G. australes* isolated from the Kermadec Islands, with strains producing a wide range of concentrations from 3–36 pg/cell, as well as producing 44-MG [12]. This is consistent with results from strains of *G. australes* isolated from the Cook Islands, which also showed no CTX but the presence of MTX-1 and 44-MG (presented as MTX-3) by LC-MS/MS [38,93]. These strains were toxic to mice, although toxicity by the i.p route of administration far exceeded that by gavage. These results are consistent with a lack of CTXs [38]. The exception to these results is one *G. australes* strain isolated from the Cook Islands, which showed CTX-activity (0.04 pg P-CTX-1 eq/cell) using the N2a bioassay although no CTXs were found by LC-MS/MS [45]. CTX-activity has also been demonstrated in *G. australes* strains isolated from elsewhere in the world (Japan, Spain, French Polynesia) using the MBA, N2a bioassay, and the RBA [19,23,64,94], but to date no CTX analogues have been reported by LC-MS/MS analysis.

Other *Gambierdiscus* and *Fukuyoa* species present in New Zealand waters include *G. honu*, which was isolated from the Kermadec Islands, and *Fukuyoa paulensis*, which was isolated from Northland’s Bay of Islands. Strains from both species were shown to produce 44-MG only by LC-MS/MS but were toxic to mice. The LD_50_ of these strains was far greater (lower toxicity) by gavage compared to i.p. injection, which is consistent with a lack of CTXs [38].

In areas of geographical significance to New Zealand, additional *Gambierdiscus* species have been detected. From the Great Barrier Reef, Australia, several species have been identified, including *G. carpenteri* Kibler, Litaker, M.A.Faust, W.C.Holland, Vandersea, & P.A.Tester, an isolate identified morphologically as *G.* cf. *belizeanus* [75], *G. honu* [39], *G. lapillus* [60], and the newly described *G. holmesii* Kretzschmar, Larsson, Hoppenrath, Doblin, & Murray and *G. lewisii* Kretzschmar, Larsson, Hoppenrath, Doblin, & Murray [79]. *Gambierdiscus carpenteri* has also been isolated from the temperate waters of Merimbula in southern New South Wales. *Gambierdiscus lapillus* strains were toxic to mice and showed both CTX and MTX activity using the FLIPR assay, despite the fact that only 44-MG was detected by LC-MS/MS, suggesting the presence of uncharacterised toxins [60,79]. Australian isolates of *G. carpenteri*, *G. holmesii*, and *G. lewisii* also produced 44-MG [73]. Although *G. lapillus*, *G. lewisii*, and *G. holmesii* all exhibited CTX- and/or MTX-like activities in bioassays, the producer of CTXs, and thus the cause of CFP in Australian waters, is still unknown. From the Cook Islands, isolates of *G. cheloniae* and *G. pacificus* were both toxic to mice, despite the presence of only 44-MG, and are being further investigated [38,43].

## 6. In Vivo Toxicity of Pure Compounds Involved in CFP

Assessment of the risk posed by any food product requires two pieces of information: the concentration of each of the contaminants in the foodstuff to allow human ingestion to be estimated and the toxicity of each individual contaminant. For CTXs this is somewhat complicated by the fact that the analogues produced by *Gambierdiscus* species are metabolized by the fish that consume them to yield additional CTX analogues, which are then consumed by humans eating the contaminated fish.

The data on the toxicity of CTXs and MTXs have been reviewed by Munday in 2014 [94]. In brief, this review reported that P-CTX-4A, P-CTX-4B, and P-CTX-3C from Pacific *Gambierdiscus* species are highly toxic to mice by intraperitoneal injection (i.p.) with LD_50_s of between 2 and 10 µg/kg. Fish metabolites, P-CTX-1 and 51-hydroxy-P-CTX-3, have also been tested in a number of studies, which have shown them to be even more toxic with i.p. LD_50_s of between 0.25 and 0.45 µg/kg. Symptoms of poisoning in mice by injection of CTXs include diarrhoea, hypersalivation, inactivity, hypothermia, and respiratory distress. There are few reported studies on the oral toxicity of CTXs, but from what is available, symptoms of intoxication are similar to those reported for i.p. injection, with the exception of the absence of diarrhoea. Potency by the two routes of administration is also reported to be similar [94].

In contrast, the water soluble MTXs are reported to be extremely potent by i.p. injection (LD_50_ as low as 0.05 µg/kg) but with an oral potency of 100 times less [94]. However, the data on which this statement is based are elusive and the oral toxicity of this class of compound should be re-evaluated.

In 2010, the EFSA CONTAM panel proposed a regulatory limit of 0.01 µg of P-CTX-1 equivalents per kg of fish [95]. To be able to determine P-CTX-1 equivalents, the panel also specified toxicity equivalence factors (TEFs) for the other CTX analogues implicated in CFP. However, the panel also noted the lack of toxicological data currently available.

## 7. Potential Uptake of CTXs and MTXs by Fish

CFP occurs following the consumption of CTX-contaminated finfish, and in the USA it is the highest reported food-borne illness attributed to fish. Twelve congeners and/or isomers of Caribbean and Atlantic-derived CTXs have been reported [96], and twenty-one congeners have been reported from Pacific-caught fish using fast atom bombardment tandem mass spectrometry (FAB-MS/MS) [97,98]. Based on the analysis of information gleaned from known cases, and applying a 10-fold safety factor, an industry and consumer advisory level of 0.10 ppb C-CTX-1 eq. has been suggested for fish from the tropical Atlantic–Gulf of Mexico–Caribbean region and 0.01 ppb P-CTX-1B eq. for the Pacific region [96].

The risk of finfish in Aotearoa/New Zealand sequestering CFP toxins was assessed by demonstration of MTX-1 uptake by Australasian snapper (*Pagrus auratus*, family Sparidae), using yellow-eyed mullet (*Aldrichetta forsteri*, family Mugilidae) inoculated with *G. australes.* It was demonstrated that, despite being a water-soluble toxin, MTX-1 can accumulate in the tissues of snapper, including the muscle [99]. Uptake was also demonstrated by the detection of *G. australes* DNA in the snapper viscera samples. A further experiment to determine the uptake of CTXs by snapper is underway.

A similar experiment was carried out using gel-food embedded with cells of *G. polynesiensis* [100] and demonstrated the uptake of CTXs into tissues by coral reef fish (in this case *Naso brevirostris*, family Acanthuridae). The results suggested that slower growing fish may accumulate higher CTX concentrations in their flesh than fast growing fish, with implications for seafood safety risk assessments.

There is still a need for rapid, sensitive assays for CTXs in fish and various approaches are being tested. For example, a cell-based assay using mammalian cardiomyoblast H9c2(2-1) cells has shown promise and will be explored further [101]. A fluorescent sandwich enzyme-linked immunosorbent assay (ELISA) has also been developed, which can differentiate and quantify four major CTX congeners at the FDA guidance level (0.01 pg/mL) [101].

## 8. Potential Impacts of Climate Change

The likely health impacts of climate change for New Zealanders were summarised by the Royal Society of New Zealand in 2017 (https://www.royalsociety.org.nz/assets/documents/Report-Human-Health-Impacts-of-Climate-Change-for-New-Zealand-Oct-2017.pdf) and include the potential for the establishment of *Gambierdiscus* in northern coastal waters with the consequent risk of occurrence of CFP in Aotearoa/New Zealand. CFP-causing dinoflagellates have been, unusually, recorded in temperate regions in southeastern Australia in recent years, indicating a potential risk of CFP toxins in these cooler ecosystems as well [25] (https://climatechange.environment.nsw.gov.au/About-climate-change-in-NSW).

Globally, the geographic regions in which *Gambierdiscus* has been reported are increasing, although partly due to increased research and sampling efforts. The genus is now known throughout the Pacific, the Caribbean, the Mediterranean, and the eastern Atlantic Ocean, as well as the Indian Ocean. CFP has also been reported from such new sites as the Canary Islands, Japan, and the Western Gulf of Mexico [10,22]. The global nature of CFP is therefore raising concerns as to the possible impacts of climate change. An analysis of a database of reported fish poisonings in the South Pacific showed a strong correlation between annual incidence of CFP and local warming of sea surfaces a decade ago. The CFPs were linked to El Niño conditions, again pointing to potential increases in CFP due to climate warming [102].

New Zealand has a small land mass, and air temperatures are highly correlated with ocean temperatures, although interannual variability occurs due to the El Nino/Southern Oscillation [103]. Rising sea temperatures are of concern and raised averaged sea surface temperatures have now been recorded for some years [104,105]. In the austral summer of 2017/2018, sea surface temperature anomalies reached +3.7 °C in the eastern Tasman Sea, exacerbated by reduced upper ocean mixing [29]. A long-term study of overlapping datasets compiled since 1981, with datasets showing good agreement, indicate significant surface warming of subtropical waters over that time. The greatest warming was to the west of New Zealand (east of Australia) and in the central Pacific, but all New Zealand coastal waters are warming [104] and this will favour *Gambierdiscus* and *Fukuyoa* growth.

Worryingly, coastal acidification in New Zealand is outpacing mean oceanic acidification. The lowest coastal pHs also tend to co-occur with nutrient loading, which has implications for the habitats of benthic dinoflagellates, although phytoplankton appear relatively resilient [106]. Ocean acidification is driven primarily by atmospheric CO_2_, but there is considerable spatial and temporal variation in pH and the carbonate system. For example, the pH of coastal waters is more variable due to such interacting factors as temperature, biological uptake and respiration, terrestrial run-off, and pollution. Calcifying organisms are particularly vulnerable to the impacts of ocean acidification and coralline algae such as *Jania* species, which are a common habitat for *Fukuyoa* in Northland, are particularly vulnerable to reduced pH [103]. Whether the changing habitat will inhibit or favour *Gambierdiscus* and *Fukuyoa* growth in the future remains to be seen.

Coral species, another favoured habitat, are an important component of benthic communities in Rangitāhua/Kermadec Islands and their response may also be significant. The future risk of CFP in New Zealand is currently an unknown, but in response to these projected changes, regular monitoring of New Zealand’s northern coastal waters is being undertaken to determine whether *Gambierdiscus* and *Fukuyoa* will bloom in the changing environment. This is already the case for the epiphytic *Ostreopsis* cf. *siamensis*, which produces palytoxin-like compounds and which now blooms most years along the northeastern coastline from the Bay of Islands in Northland to Leigh, further south in the Hauraki Gulf [16]. The data indicate that nothing is simple, and while there is the real possibility that warming waters will favour *Gambierdiscus* growth (as indicated by the correlation between the Southern Oscillation Index and increased CFP cases), large bodies of extremely warm water, which last for extended periods, as in the Indo-Pacific Warm Pool, may depress CFP rates [107]. To complicate things even further, quorum-sensing bacteria have been shown to impact the growth and toxin production of *Gambierdiscus* [107], and the effects of climate change on associated bacterial species, and thus on CFP, can only be speculated on at this time.

## 9. Conclusions

Known CTX/MTX producers in Aotearoa/New Zealand’s coastal and territorial waters have increased over the last decade (Figure 1) [11,18,39] and, as New Zealand’s coastal waters warm, the likelihood of the CTX-producing *G. polynesiensis* being detected in northern New Zealand increases. Monitoring for *Gambierdiscus* in mainland New Zealand waters using artificial samplers will continue with samples analysed using a combination of high-throughput sequencing techniques (for example, metabarcoding, which reveals high species richness) [35] and qPCR approaches to determine the presence or absence of species in the genus. A focus will be on sites that are a known habitat for *Fukuyoa* in the Bay of Islands. These sites are also close to Port Opua, the entry point for yachts and recreational craft arriving from international waters, and which are potential vectors for the introduction of new *Gambierdiscus* species to the region.

Climate-driven changes in biotic interactions could have a dramatic impact on coral communities, which can shift to macroalgal forests if fish herbivory decreases. In temperate regions, a potential shift from macroalgae to ‘barrens’ can occur if tropical herbivore numbers increase. If oceanic hot spots, as occurred in the Tasman Sea in 2017/2018 [29], are circulated globally this ‘tropicalisation’ of temperate marine ecosystems could occur in New Zealand [30], with impacts on benthic and epiphytic HAB development and CFP risk. The recent focus on collating data on the distribution of HAB species, and in particular *Gambierdiscus*, through HABMAP (a subsection of the IOC-UNESCO Ocean Biogeographic Information System) [5], will give some insights into the likelihood of specific species occurring in New Zealand waters, and thus the potential for CFP toxins to accumulate in local fish populations in the future.

## Figures and Tables

**Table 1:** Selected strains of species in the dinoflagellate genera *Gambierdiscus* and *Fukuyoa* isolated from Te Uenga Bay, Northland, New Zealand and from North Meyer and Macauley Islands, Rangitāhua/Kermadec Islands: Geographic distribution and toxin production.

Species	Sampling Site	GenBank Code	Toxins (pg/cell)	CICCM Code	Reference
*G. australes*	North Meyer Is. 29°14.485′ S, 172°52.717′ W	KY069059	MTX-1 (5.9); 44-MG	CAWD244	Rhodes et al. 2017 [37]
	North Meyer Is. 29°14.485′ S, 172°52.717′ W	MN709498	MTX-1 (8.9); 44-MG	CAWD246	Munday et al. 2017 [38]
	Macauley Is. 30°14′ S, 178°26′ W	MF109033	MTX-1 (36); 44-MG	CAWD255	Rhodes et al. 2017 [12,37]
	Macauley Is. 30°14′ S, 178°26′ W	MF109034	MTX-1 (31); 44-MG	CAWD256	Rhodes et al. 2017 [12]
*G. honu*	North Meyer Is. 29°14.485′ S, 172°52.717′ W	KY062662	44-MG	CAWD242	Rhodes et al. 2017 [39]
*G. polynesiensis*	Macauley Is. 30°14′ S, 178°26′ W	MF109032	Traces CTX-3C; iso peaks CTX-3B/C and 4A/B; 44-MG	CAWD254	Unpublished data
	Macauley Is. 30°14′ S, 178°26′ W	NE	Neg. CTXs, MTX, 44-MG	CAWD259	Rhodes et al. 2017 [12]
*G.* cf. *toxicus* *	Northland	NS	NT		Chang 1996 [18]
*F. paulensis*	Te Uenga Bay, Northland 35°25.58′ S, 174°24.17′ E	MN305995	44-MG	CAWD306	Unpublished data

*: *Gambierdiscus toxicus* was reclassified in 2009 by Litaker et al. [36], therefore toxins published from this species may differ from that reported prior to that time.

**Table 2:** Toxin production profiles for known *Gambierdiscus* and *Fukuyoa* species.

Species	Toxins	Toxicity	Reference
*G. australes*	MTX-1, 44-MG	High toxicity by i.p. using the MBA, less by oral administration	Munday et al. 2017 [38]
*G. balechii*	44-MG	CTX- and MTX-like toxicity using the MBA and the N2a cytotoxicity assay	Fraga et al., 2016 [74]; Pisapia et al., 2017 [23]
*G. belizeanus*	Gambierone, 44-MG	CTX-like toxicity using the N2a cytotoxicity assay; CTX- and MTX-like activity using the Ca^2+^ flux assay; CTX-like activity using the RBA	Chinain et al. 2010 [19]; Rodriguez et al. 2015 [75]; Lewis et al. 2016 [76]; Litaker et al. 2017 [22]; Boente-Juncal et al. 2019 [77]
*G. caribaeus*	MTX-2, 44-MG	CTX- and MTX-like toxicity using the N2a cytotoxicity assay; CTX- and MTX-like activity using the MBA	Tawong et al. 2016 [78]; Litaker et al. 2017 [22]; Pisapia et al. 2017 [23]
*G. carolinianus*	44-MG	Extremely low CTX- and MTX-like toxicity using the N2a cytotoxicity and Ca^2+^ flux assays	Tester et al. 2014 [51]; Lewis et al. 2016 [77]; Litaker et al. 2017 [22]; Pisapia et al. 2017 [23]
*G. carpenteri*	44-MG*	Low toxicity by i.p. using the MBA, less by oral administration; CTX- and MTX-like toxicity using the N2a cytotoxicity assay; MTX-like activity using the Ca^2+^ flux assay	Munday et al. 2017 [38]; Litaker et al. 2017 [22]; Pisapia et al. 2017 [23]; Larsson et al. 2018 [26]
*G. cheloniae*	44-MG	High toxicity by i.p. using the MBA, less by oral administration	Smith et al. 2016 [44]; Munday et al. 2017 [38]
*G. excentricus*	MTX-2, MTX-4, 44-MG	High CTX- and MTX-like toxicity using the N2a cytotoxicity and Ca^2+^ flux assays	Fraga et al. 2011 [21]; Pisapia et al. 2017 [23]
*G. holmesii*	44-MG	CTX- and MTX-like activity using the Ca^2+^ flux assay	Larsson et al. 2018 [26]; Kretzschmar et al. 2019 [79]
*G. honu*	44-MG	High toxicity by i.p. using the MBA, less by oral administration	Munday et al. 2017 [38]; Rhodes et al. 2017 [39]
*G. jejuensis*	Unknown	Unknown (non-toxic by i.p. using MBA)	Nishimura et al. 2014 [64]
*G. lapillus*	44-MG	CTX- and MTX-like activity using the Ca^2+^ flux assay	Kretzschmar et al. 2017 [60]; Larsson et al. 2018 [26]
*G. lewisii*	44-MG	CTX- and MTX-like activity using the Ca^2+^ flux assay	Larsson et al. 2018 26]; Kretzschmar et al. 2019 [79]
*G. pacificus*	44-MG, MTX-2	High toxicity by i.p. using the MBA, less by oral administration, CTX- and MTX-like activity using the N2a cytotoxicity assay	Munday et al. 2017 [38]; Pisapia et al. 2017 [23]
*G. polynesiensis*	P-CTX-3B *, P-CTX-3C *, P-CTX-4A *, P-CTX-4B *, P-CTX-3B/C isomers *, P-CTX-4A/B isomers *, 44-MG	Highly toxic by both i.p. and oral administration using the MBA	Rhodes et al. 2014 [33]; Munday et al. 2017 [38]; Rhodes et al. 2017 [12]; Chinain et al. 2010 [19]
*G. scabrosus*	44-MG	CTX- and MTX-like toxicity using the MBA and N2a cytotoxicity assay	Nishimura et al. 2014 [64,80]; Pisapia et al. 2017 [23]
*G. silvae*	44-MG	CTX- and MTX-like toxicity using the N2a cytotoxicity assay	Litaker et al. 2017 [22]; Pisapia et al. 2017 [23]
*G. toxicus* ^†^	P-CTX-3C, P-CTX-4A/B, 44-MG, Gambieric acids A, B, C, and D, Gambieroxide, Gambierol, MTX-1, MTX-2	CTX- and MTX-like toxicity using the N2a cytotoxicity and RBA assays; MTX-like activity using the MBA	Holmes et al. 1990 [81]; Nagai et al. 1992 [82]; Satake et al. 1993 [83]; Watanabe et al. 2013 [84]; Pisapia et al. 2017 [23]
*F. paulensis*	44-MG	Low toxicity by i.p. using the MBA, extremely low by gavage	Rhodes et al. 2014 [32]; Munday et al. 2017 [38]
*F. ruetzleri*	44-MG *	CTX-like toxicity using the N2a cytotoxicity, Ca^2+^ flux and brine shrimp assays	Tester et al. 2014 [51]; Litaker et al. 2017 [22]; Leung et al. 2018 [85]
*F. yasumotoi*	Unknown	MTX-like activity using the MBA	Holmes 1998 [86]
*Fukuyoa* sp. HK Type 1	44-MG	Activity using the brine shrimp bioassay	Leung et al. 2018 [85]

i.p: intraperitoneal injection; MBA: mouse bioassay; RBA: receptor binding assay; N2a: neuro-2 mouse neuroblastoma assay; CTX: ciguatoxin; MTX: maitotoxin; 44-MG: 44-methylgambierone; *: toxins asterisked were not produced by all strains of these species. ^†^ *Gambierdiscus toxicus* was reclassified in 2009 by Litaker et al. [36], therefore toxins published from this species may differ from that reported prior to that time.
